# Platelet-Based Blood Products in Bovine and Equine Medicine: A Systematic Scoping Review of Blood Collection, Preparation Methods, and Reporting Completeness

**DOI:** 10.3390/vetsci13101112

**Published:** 2026-10-18

**Authors:** Filippo Rigo, Caterina Marcon, Margherita Soncin, Flaviana Gottardo

**Affiliations:** 1 Department of Animal Medicine, Production and Health, University of Padua, 35020 Legnaro, Italy; caterina.marcon@unipd.it; 2 Istituto Zooprofilattico Sperimentale delle Venezie, 35020 Legnaro, Italy; msoncin@izsvenezie.it

**Keywords:** platelet-rich plasma, platelet lysate, platelet concentrate, bovine, equine, blood collection, methodological reporting, scoping review, mastitis

## Abstract

Platelet-based blood products, including platelet-rich plasma, platelet concentrate, and platelet lysate, are increasingly investigated in veterinary medicine as biological preparations with proposed regenerative, anti-inflammatory, and immunomodulatory properties. In bovine and equine medicine, their use has expanded across several clinical fields, but blood collection and product preparation methods have not been systematically synthesized. This systematic scoping review aimed to describe the literature on platelet-based blood products in bovine and equine medicine, summarize methodological characteristics of blood collection and preparation methods, and analyze studies addressing bovine mammary disorders. Searches were performed in Scopus, Web of Science Core Collection, CAB Abstracts, and PubMed on 25 August 2026, with no publication-year or start-date restriction. Eligible studies were original peer-reviewed articles in English; fibrin- or gel-based preparations were excluded when the corresponding liquid platelet-based product could not be separately characterized or evaluated, as were scaffold or combined constructs when the platelet-based component could not be evaluated separately. No formal review protocol was registered or published. Studies providing eligible bovine and/or equine data and reporting at least one methodological aspect related to collection, preparation, activation or lysis, storage, characterization, or administration were eligible. A total of 191 studies were included. Studies contributing equine data only among the target species predominated (161/191; 84.3%), whereas 29 studies (15.2%) contributed bovine data only and one study (0.5%) included both bovine and equine data. The PRP family, including PRP and related PRP-type preparations, was the most frequently investigated product group (134/191; 70.2%), followed by platelet lysate (46/191; 24.1%) and platelet concentrate (19/191; 9.9%); categories were not mutually exclusive. Methods varied in donor source, collection site, blood volume, anticoagulant, collection system, centrifugation workflow, activation or lysis, storage, and characterization. The main blood collection and preparation workflow was reconstructable in 111 studies (58.1%) and partially reconstructable in 80 studies (41.9%). Methodological reporting was frequently incomplete, particularly for leukocyte concentration, activation or lysis procedures, storage conditions, and product-level sterility or microbiological quality control. Product-level sterility or microbiological quality control was explicitly reported in 7 studies (3.7%) and partially reported in one study (0.5%). Only five studies addressed bovine mammary disorders and investigated methodologically distinct products. More complete and consistent methodological reporting is needed to improve reproducibility and comparability across studies.

## Simple Summary

Platelet-based blood products are biological preparations obtained from blood and are increasingly studied in veterinary medicine because they have been suggested to contribute to tissue repair and inflammatory modulation. They are used in horses and cattle for different conditions, including joint and tendon problems, reproductive disorders, wounds, and udder diseases. However, these products are not all prepared in the same way. Differences in how blood is collected, processed, stored, and administered can change the final product and may affect its biological activity and clinical results. This review examined 191 studies on platelet-based blood products in bovine and equine medicine, with special attention to how these products were prepared and reported. Only peer-reviewed studies published in English were included. Fibrin- or gel-based products, including platelet-rich fibrin, and scaffold or combined preparations were excluded when the liquid platelet-based component could not be evaluated separately. No formal review protocol was registered or published. The review showed that most studies were performed in horses, while fewer studies involved cattle. The PRP family, including PRP and related PRP-type preparations, was the most frequently investigated product group, although platelet lysate and platelet concentrates were also represented. Only five studies addressed bovine mammary disorders. Across the literature, preparation methods and reporting completeness varied widely, especially for leukocyte content, activation or lysis procedures, storage conditions, and microbiological quality control. The main preparation workflow was reconstructable in 111 studies (58.1%), while 80 studies (41.9%) provided only a partially reconstructable workflow. These findings suggest that future studies should describe preparation methods more clearly and follow more consistent reporting standards. More consistent reporting may help researchers and veterinarians compare results, reproduce protocols, and evaluate the potential clinical value of these products more reliably.

## 1. Introduction

Platelet-based blood products, including platelet-rich plasma (PRP), platelet concentrates (PC), platelet lysate (PL), and related products, are increasingly investigated in veterinary medicine as biological products with proposed regenerative, anti-inflammatory, and immunomodulatory properties [1,2]. In this review, the umbrella term “platelet-based blood products” is used for these preparations to avoid the term “hemoderivatives”, which may have a narrower regulatory meaning referring to industrially manufactured plasma-derived medicinal products. Their use has expanded across several clinical fields, particularly in large-animal medicine, where they have been explored for musculoskeletal disorders, reproductive diseases, wound healing, cell-based and methodological applications and, more recently, mammary conditions [3,4,5].

Despite this growing interest, interpretation of the available evidence remains challenging because platelet-based blood products are not standardized preparations. Studies differ widely in donor selection, blood collection site and volume, anticoagulants, collection systems, centrifugation protocols, activation or lysis procedures, storage conditions, and final product characterization. Consequently, products grouped under the same label, such as PRP, PC, or PL, may differ markedly in cellular composition, concentration of platelet-derived mediators, leukocyte content, and potential biological activity [1,2,6,7]. This variability is compounded by incomplete methodological reporting, which may prevent full reconstruction of the preparation workflow even when the general product category can be identified. This methodological variability limits comparability among studies and complicates reproducibility, interpretation of clinical outcomes, and translation into field practice.

These issues are particularly relevant in bovine and equine medicine. In these species, blood collection and product preparation may be performed under farm, field, or ambulatory conditions, where practical constraints related to animal handling, sterility, sample transport, processing time, blood volume availability, and equipment access may directly affect the feasibility and standardization of platelet-based blood product preparation [4,8]. Equine medicine has generated a larger body of literature, especially in musculoskeletal applications, but this literature also highlights substantial heterogeneity in preparation methods and incomplete characterization of final products as major limitations [4,9]. In contrast, bovine studies remain more limited and fragmented, despite increasing interest in reproductive and mammary applications [5]. At the same time, recent expansion of the literature indicates that platelet-based blood products are being investigated in an increasingly diverse range of experimental and clinical settings, making consistent product definition and methodological reporting even more important.

Previous reviews have addressed platelet concentrates in bovine practice [5] or critically summarized PRP use in equine medicine [4], but neither was designed to systematically compare methodological reporting across both species and across multiple platelet-based product families. The present review extends this previous work by jointly mapping 191 bovine and equine studies, explicitly evaluating workflow reconstructability and reporting completeness across predefined methodological domains, and examining product-level sterility or microbiological quality-control reporting. This broader methodological focus allows comparison of reporting practices across species, product families, study designs, and publication periods rather than concentrating primarily on clinical applications or a single product category.

Within bovine medicine, mammary disorders deserve particular attention. Mastitis remains one of the most prevalent and economically important diseases in dairy cattle worldwide and is one of the major drivers of antimicrobial use in dairy practice [10,11,12,13]. In this context, platelet-based blood products have been proposed as potential complementary or antimicrobial-sparing approaches for intramammary use. However, the number of studies specifically addressing bovine mammary disorders remains small, and the available evidence is methodologically heterogeneous, with differences in donor selection, blood collection procedures, product preparation, treatment protocols, comparators, and outcome assessment [14,15,16,17,18]. Moreover, the available studies have investigated technically distinct platelet-based preparations rather than repeated applications of a single standardized intervention, which further limits direct comparison of their clinical findings.

Understanding how these products are collected, processed, characterized, stored, and administered is therefore important before their biological rationale and clinical results can be meaningfully interpreted. This is especially true for products such as PRP, PC, and PL, which should not be considered interchangeable interventions when they are derived through different workflows or incompletely characterized [1,2]. A structured methodological synthesis may therefore help clarify the extent of procedural variability across studies, identify recurrent reporting gaps, and identify core information that should be reported to improve reproducibility in future research. Such an approach is particularly relevant in a field where increasing publication volume does not necessarily correspond to greater methodological comparability.

The present review was developed as the methodological foundation for a broader research line investigating platelet-based blood products for bovine mammary applications, with particular interest in their possible role as complementary or antimicrobial-sparing approaches in dairy cattle. During the development of this research line, it became necessary to clarify how platelet-based blood products have been obtained, characterized, stored, and administered in large-animal medicine, and to determine whether studies addressing bovine mammary disorders could be meaningfully compared. Although reproductive, musculoskeletal, wound-healing and other experimental or methodological applications were included in the descriptive and methodological synthesis, a focused analysis of mammary applications was included as a specific review objective because of their potential relevance as complementary or antimicrobial-sparing approaches in dairy practice. Therefore, the aims of the present systematic scoping review were (i) to provide a descriptive overview of studies investigating platelet-based blood products in bovine and equine medicine; (ii) to systematically summarize the methodological characteristics of blood collection, product preparation, product characterization, storage, and administration across included studies; and (iii) to perform a focused analysis of studies addressing bovine mammary disorders, in order to describe the methodological variability that should be considered when comparing and interpreting these studies.

## 2. Materials and Methods

### 2.1. Review Design

The present study was designed as a systematic scoping review aimed at describing the methodological characteristics of blood collection and platelet-based blood product preparation in bovine and equine medicine, with a focused analysis of studies addressing bovine mammary disorders. The review was conducted according to the JBI methodological guidance for scoping reviews and was reported in accordance with the PRISMA-ScR checklist [19,20]. No formal review protocol was registered or published.

### 2.2. Search Strategy

Searches were conducted in Scopus, Web of Science Core Collection, CAB Abstracts, and PubMed on 25 August 2026. No publication-year or start-date restriction was applied; searches covered all records indexed in each database up to the search date. Search strategies were adapted to the syntax and indexing structure of each database and were based on combinations of terms related to platelet-based blood products, including platelet-rich plasma (PRP), platelet concentrate (PC), platelet lysate (PL), and related expressions, combined with species-specific terms for bovine and equine subjects. Search terms intentionally included platelet-rich fibrin (PRF) and related terminology to maximize sensitivity. However, fibrin- or gel-based preparations were outside the analytical scope of the review when the corresponding liquid platelet-based product could not be separately characterized or evaluated. No search-level exclusion terms were applied. Database-specific filters were used only where appropriate, including the English-language filter in CAB Abstracts. Species eligibility and study relevance were subsequently determined during title/abstract and full-text screening. The complete database-specific search strings, including search terms and filters, are reported in Supplementary Table S1. The searches retrieved 1729 records from Scopus, 2062 from Web of Science Core Collection, 388 from CAB Abstracts, and 1380 from PubMed.

After completion of the full-text screening process, the reference lists of relevant review articles and included studies were examined to identify potentially eligible primary studies not captured by the database searches. No additional eligible studies were identified through reference checking.

### 2.3. Eligibility Criteria

Studies were eligible for inclusion if they met all of the following criteria: (i) provided data pertaining to bovine and/or equine subjects that could be separately identified and extracted; (ii) investigated, described, or applied a platelet-based blood product; (iii) provided sufficient information to classify the platelet-based product and to extract at least one methodological variable relevant to blood collection, preparation, activation or lysis, storage, characterization, or administration; and (iv) were original peer-reviewed studies. Studies including additional non-target species were retained when the eligible bovine or equine data could be separately identified and extracted.

Studies were excluded if they (i) did not provide separately identifiable bovine or equine data; (ii) were review articles, editorials, conference abstracts without sufficient methodological information, or other non-original publications; (iii) focused on products unrelated to the platelet-based blood products within the scope of this review; (iv) lacked information relevant to the methodological aims of the review; (v) were single case reports or purely descriptive case series that did not meet the specified study-design requirements; (vi) focused exclusively or primarily on platelet-rich fibrin, platelet-derived gels, or other fibrin-based preparations when the corresponding liquid platelet-based preparation could not be separately characterized or evaluated; (vii) were not written in English; or (viii) had no full text available.

Studies were retained when PRP, PC, PL, or another eligible platelet-based blood product was independently characterized or evaluated, even if gel formation or a gel-based condition was also investigated.

Studies in which PRP or another platelet-based blood product was used only as a component of a combined scaffold or tissue-engineering construct were excluded when the platelet-based product could not be evaluated as a distinct intervention. Studies were retained when the platelet-based preparation was separately characterized, manipulated, or evaluated as an independent experimental component or across distinct experimental conditions.

For the focused bovine mammary subset, studies were additionally classified as relevant when they addressed mastitis, intramammary treatment, mammary gland disorders, udder disease, or teat-related conditions.

### 2.4. Study Selection

The selection process was conducted sequentially after completion of the database searches. Records retrieved from Scopus, Web of Science Core Collection, CAB Abstracts, and PubMed were exported, combined into a single reference dataset, and imported into Rayyan, where duplicate records were identified and removed. The remaining records were then screened by title and abstract. Subsequently, the full texts of potentially eligible studies were retrieved and assessed against the review eligibility criteria. Screening and eligibility assessment were conducted using Rayyan (https://www.rayyan.ai/, accessed on 17 October 2026) [21]. Title and abstract screening and full-text eligibility assessment were performed independently by two reviewers (F.R. and C.M.) using the eligibility criteria described above. Disagreements were resolved through discussion until consensus was reached. When eligibility could not be determined from title and abstract alone, the record was retained for full-text assessment. Full-text screening was used to confirm final eligibility. Reports were excluded at this stage when the full text was not in English, when the platelet-based blood product was used only as part of a scaffold or combined construct without a separately evaluable effect, when the report was a purely descriptive case series that did not meet the specified study-design requirements, when it was a conference abstract or report with insufficient methodological information, when it was a non-journal publication, or when it represented a duplicate report of a study already included in the review. When full texts were not immediately available through institutional access, additional retrieval attempts were made through library resources, publisher websites, and other institutional sources before exclusion. Duplicate reports identified during study selection were not counted as independent studies. The eligibility criteria applied during screening were not modified after screening began. The more detailed wording concerning fibrin- or gel-based products, scaffold or combined constructs, and study-design eligibility reflects clarification of the criteria applied during screening and did not alter the final study set.

### 2.5. Data Charting and Reconstructability

Data were extracted using a structured charting form developed specifically for this review. The charting form was pilot-tested on a subset of included studies and then refined before full data extraction. Data extraction was performed by one reviewer (F.R.) and subsequently verified by a second reviewer (C.M.) using the same standardized charting form. The second reviewer had access to the initial extraction during verification. Discrepancies or uncertain classifications were resolved through discussion between the two reviewers until consensus was reached. Before final synthesis, the complete study-level dataset was systematically reassessed across all 191 included studies to ensure consistent application of the operational definitions used for study type, product-family assignment, workflow reconstructability, and domain-level reporting. This reassessment concerned the analytical classification of the existing study-level extraction and did not alter the set of included studies. When classifications were uncertain or inconsistent, the corresponding study information was re-examined before the final category was assigned.

Data extraction was conducted in two sequential levels. First, all included studies underwent full-text extraction for core descriptive and methodological variables. For each study, the following information was collected when available: bibliographic information, publication year, species, study type, clinical or application area, type of platelet-based blood product, product source, blood collection method, product preparation method, activation or lysis procedure, storage condition, product characterization, sterility or microbiological quality control, and administration route.

Following completion of study-level data extraction, an analytical classification was developed during the synthesis stage to summarize whether the main blood collection and preparation workflow could be reconstructed from the reported information. This classification was then applied systematically to all included studies using explicit decision rules. This overall assessment was distinct from the domain-specific reporting-gap analysis described below and did not imply complete reporting of every methodological item. Studies were classified as having a reconstructable main workflow when product identity, blood source or collection system, anticoagulant or an identifiable commercial or cited preparation protocol, the principal processing sequence, and final experimental or clinical use could be reconstructed from the article or from an explicitly identifiable cited or commercial protocol. Studies were classified as having a partially reconstructable main workflow when one or more of these core elements could not be recovered with sufficient certainty. Missing secondary numerical details, such as exact blood volume or individual centrifugation parameters, did not by themselves result in classification as partially reconstructable when the overall workflow remained identifiable. For example, a study using a clearly identifiable commercial preparation system could still be classified as reconstructable even if some individual centrifugation parameters were not explicitly reported, provided that the overall preparation workflow could be identified with sufficient certainty. Conversely, a study reporting only that PRP was used, without sufficient information on the collection system or principal preparation steps, was classified as partially reconstructable. Eligibility required sufficient information to classify the platelet-based blood product and extract at least one methodological variable.

For the reporting-gap analysis, nine methodological domains were assessed: blood collection volume, collection site, anticoagulant, centrifugation force and/or time, platelet concentration, leukocyte concentration, activation or lysis method, storage condition, and sterility or microbiological quality control. These domains were selected because they represent recurring methodological components required to describe blood collection, platelet-based product preparation, product characterization, handling, and microbiological quality control across the eligible study designs. They were used as operational categories for the present review and were not derived from a previously validated reporting framework.

For the bovine mammary subset, data extraction was performed in greater detail to allow direct comparison among studies regarding clinical condition, experimental unit and sample size, product type and source, preparation workflow, dose and administration schedule, comparator treatments, allocation and blinding when reported, follow-up and outcome definitions, and product-level sterility or microbiological quality control.

### 2.6. Data Categorization

For descriptive synthesis, included studies were categorized according to the variables described below. Publication year was grouped into four periods: ≤2014, 2015–2018, 2019–2022, and 2023–2026. Species were classified as equine, bovine, or both bovine and equine according to the eligible target-species data contributed by each study. Studies including additional non-target species were classified according to the bovine and/or equine data eligible for this review, irrespective of the concurrent inclusion of other species. Study type was categorized as in vivo, in vitro or ex vivo, or methodological according to the experimental setting and primary study objective. For mixed designs, an in vivo component took precedence. In the absence of an in vivo component, studies evaluating a downstream biological effect or function of the platelet-based product in cells, tissues, microorganisms, gametes, embryos, or other ex vivo/in vitro systems were classified as in vitro/ex vivo. Studies whose primary objective concerned product collection, preparation, processing, storage, characterization, or quality control, rather than downstream biological effects, were classified as methodological. Clinical or application area was classified as mammary, reproductive, musculoskeletal, wound healing, methodological, or other. For graphical presentation of clinical or application areas, methodological studies were excluded and related clinical areas were grouped where appropriate.

The terminology used by the original authors to describe each platelet-based blood product was retained during study-level data extraction. For descriptive synthesis, preparations were subsequently grouped into three main product families: PRP and related PRP-type preparations, platelet lysate (PL), and platelet concentrate (PC). Specific preparations such as pure platelet-rich plasma (P-PRP), autologous conditioned plasma (ACP), plasma rich in growth factors (PRGF), freeze-dried PRP, and other related PRP-type preparations were retained in the study-level dataset but were aggregated within the broader PRP family when appropriate for summary analyses. Product-family assignment was based on the distinct platelet-based preparation actually evaluated, characterized, or administered in each study. Upstream intermediates used only to generate a final product were not assigned as an additional product family. Freeze–thaw treatment alone did not result in reclassification of PRP or PC as PL unless the resulting lysate or platelet extract was explicitly identified and evaluated as a distinct preparation. Studies evaluating more than one product family were assigned to each relevant category; therefore, aggregate product-family categories were not mutually exclusive.

Product family was considered separately from biological source, activation or lysis, and storage or handling. Biological source was classified according to the relationship between donor and recipient as autologous, allogeneic or donor-based, mixed/comparative, or not reported/unclear, as appropriate. The use of a commercial kit or proprietary preparation system was recorded as part of the collection or preparation workflow rather than as a biological source. Activation or lysis procedures and storage or handling conditions were recorded in their respective methodological domains.

For the assessment of methodological reporting gaps, each item was classified as reported, partially reported, not reported or not applicable. “Reported” indicated that the item was explicitly described and usable for methodological comparison. “Partially reported” indicated that the item was described incompletely, indirectly, or through reference to a commercial kit or previously published protocol but remained partially interpretable. “Not reported” indicated that the methodological domain was considered applicable, but the corresponding information was absent or not recoverable from the full text. “Not applicable” was assigned only when the reported preparation workflow clearly demonstrated that the methodological item did not apply. When the available information was insufficient to determine whether the domain applied, the item was classified as “not reported” rather than “not applicable”. An explicit statement that a procedure was not performed was considered reported information rather than not applicable. For sterility or microbiological quality control, “reported” required an explicit product-level microbiological or sterility assessment, such as bacterial or fungal culture, sterility testing, mycoplasma testing, or endotoxin testing. Aseptic or sterile handling alone, diagnostic bacteriology unrelated to product quality control, and antimicrobial activity testing were not considered equivalent to product-level sterility testing. For each methodological domain, percentages of reported, partially reported, and not reported items were calculated using the number of studies for which that domain was applicable as the denominator; studies classified as not applicable were reported separately.

Items were treated as reporting domains rather than mandatory procedural steps; therefore, “not reported” indicated absence of interpretable information and did not necessarily imply that the procedure was not performed.

### 2.7. Data Synthesis

A descriptive synthesis was performed for all included studies. Frequencies and proportions were calculated for publication period, species, study type, clinical or application area, and product family. Selected methodological characteristics contributing to preparation heterogeneity, including biological source, blood collection site, anticoagulant family, activation or lysis approach, and storage or handling category, were also summarized using frequencies and proportions.

Methodological findings were summarized both narratively and in tabular format. The level of methodological reporting was first summarized across all included studies. A detailed supplementary table was prepared to document study-level information on blood collection, anticoagulant use, preparation workflows, activation or lysis procedures, storage, product characterization, sterility or microbiological quality control, and administration. Methodological reporting gaps were then summarized by calculating the proportion of studies in which key items were reported, partially reported, or not reported. To explore whether reporting completeness differed across major study characteristics, domain-level reporting was additionally stratified by species, study type, product family, and publication period. Reporting patterns were also compared between studies classified as having reconstructable and partially reconstructable workflows.

Sterility or microbiological quality-control reporting was additionally examined in subgroups considered particularly relevant from a product-safety perspective, including studies involving in vivo administration and studies using allogeneic/donor-based, pooled, or stored/frozen/freeze-dried products. These subgroup analyses described reporting frequency only and were not intended as assessments of microbiological risk or product safety.

A focused table was prepared for studies addressing bovine mammary disorders, summarizing clinical condition and sample size, platelet-based blood product and source, main preparation features, dose and administration schedule, comparator treatments, allocation and blinding when reported, follow-up and outcome definitions, and product-level sterility or microbiological quality control. Given the substantial heterogeneity in study design, intended clinical use, product type, preparation protocol, and outcome assessment, no quantitative synthesis or meta-analysis was performed. Accordingly, findings were synthesized descriptively rather than pooled across clinically and methodologically heterogeneous studies.

Data were managed using Microsoft Excel for Microsoft 365 (Microsoft Corporation, Redmond, WA, USA). Frequencies and proportions were calculated in Microsoft Excel for Microsoft 365, and figures were prepared using Microsoft Excel for Microsoft 365 and ChatGPT (OpenAI; https://chatgpt.com/, accessed on 17 October 2026)-assisted graphical refinement.

## 3. Results

### 3.1. Study Selection

**Figure 1:**
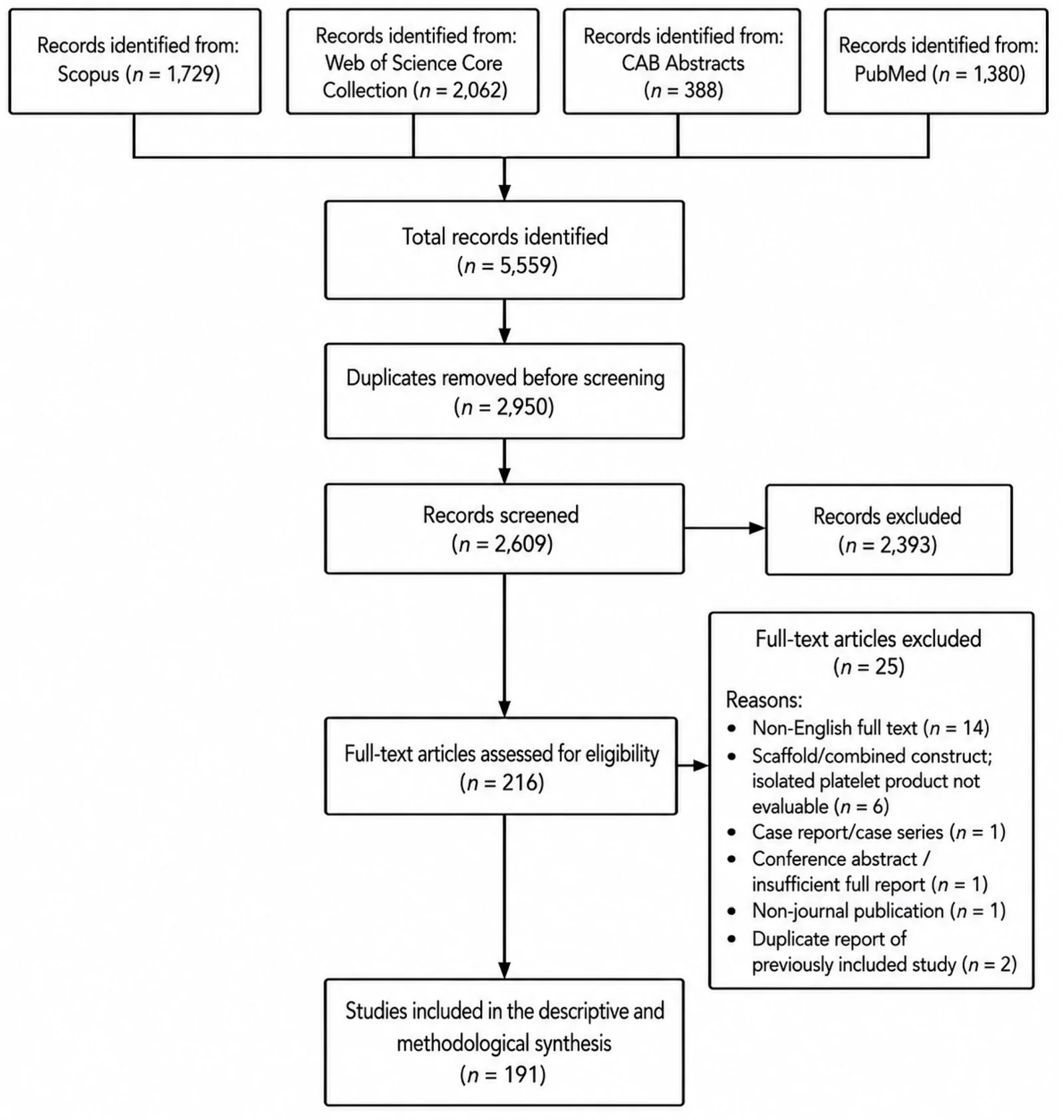
PRISMA flow diagram of the study selection process. Flow diagram showing study identification through Scopus, Web of Science Core Collection, CAB Abstracts, and PubMed, duplicate removal, title and abstract screening, full-text eligibility assessment, and final inclusion of studies in the descriptive and methodological synthesis.

The study selection process is summarized in Figure 1. A total of 5559 records were identified through database searching, including 1729 records from Scopus, 2062 from Web of Science Core Collection, 388 from CAB Abstracts, and 1380 from PubMed. After removal of 2950 duplicate records, 2609 unique records were screened by title and abstract. Of these, 2393 were excluded, and 216 full-text reports were assessed for eligibility.

Twenty-five reports were excluded at the full-text stage for the following reasons: non-English full text (*n* = 14), platelet-based blood product used only as part of a scaffold or combined construct without a separately evaluable effect (*n* = 6), descriptive case series not meeting the specified study-design requirements (*n* = 1), conference abstract or report with insufficient methodological information (*n* = 1), non-journal publication (*n* = 1), and duplicate report of a study already represented in the review (*n* = 2). The 14 reports excluded because of language comprised 11 equine and three bovine studies (nine published in Portuguese, four in Spanish, and one in German). Thirteen investigated PRP or PRP-type preparations, including ACP, and one evaluated equine platelet concentrates; the topics included preparation or product characterization, experimental musculoskeletal and cutaneous applications, and in vitro antimicrobial activity.

Therefore, 191 studies were included in the final descriptive and methodological synthesis; the included primary studies are cited in References [8,9,14,15,16,17,18,22,23,24,25,26,27,28,29,30,31,32,33,34,35,36,37,38,39,40,41,42,43,44,45,46,47,48,49,50,51,52,53,54,55,56,57,58,59,60,61,62,63,64,65,66,67,68,69,70,71,72,73,74,75,76,77,78,79,80,81,82,83,84,85,86,87,88,89,90,91,92,93,94,95,96,97,98,99,100,101,102,103,104,105,106,107,108,109,110,111,112,113,114,115,116,117,118,119,120,121,122,123,124,125,126,127,128,129,130,131,132,133,134,135,136,137,138,139,140,141,142,143,144,145,146,147,148,149,150,151,152,153,154,155,156,157,158,159,160,161,162,163,164,165,166,167,168,169,170,171,172,173,174,175,176,177,178,179,180,181,182,183,184,185,186,187,188,189,190,191,192,193,194,195,196,197,198,199,200,201,202,203,204,205].

### 3.2. Descriptive Overview of Included Studies

**Figure 2:**
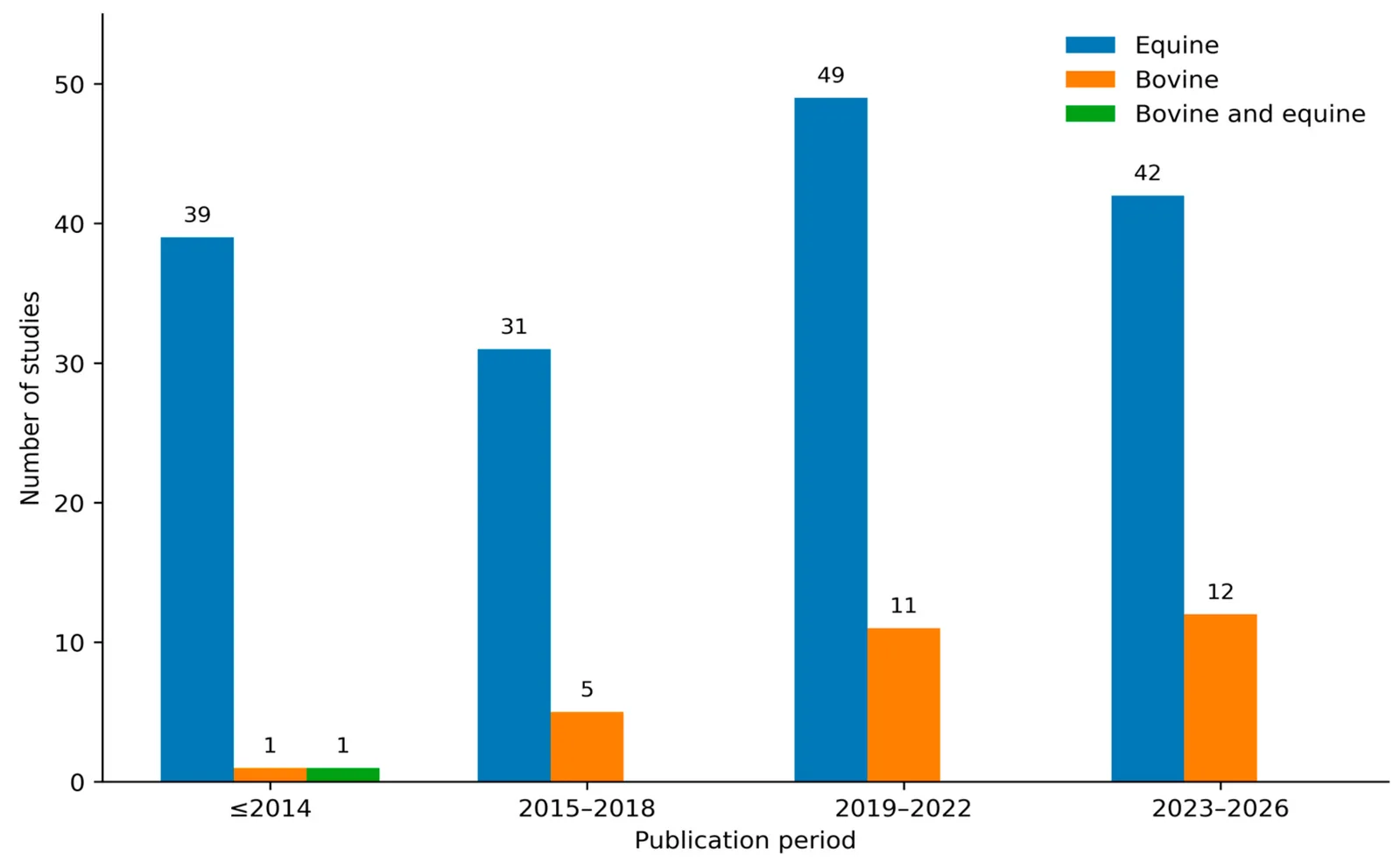
Distribution of included studies by publication period and target species. Bar chart showing the number of included studies across four publication periods, classified according to the eligible target-species data contributed by each study as equine, bovine, or both bovine and equine.

**Figure 3:**
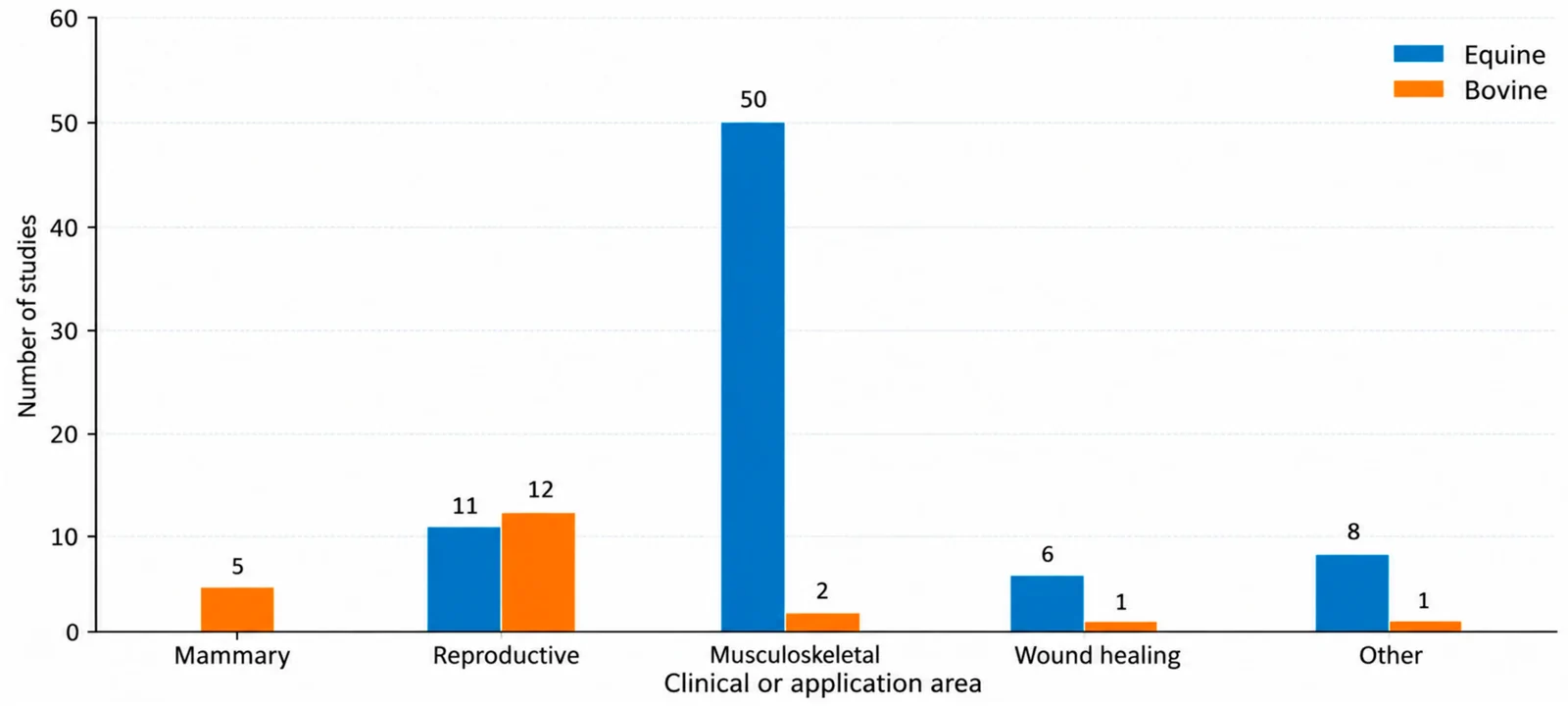
Distribution of studies with a clearly classified clinical or application area according to target species. Methodological/product-characterization studies and studies for which a specific clinical or application area could not be assigned were not included.

The descriptive characteristics of the 191 included studies are reported in Table 1. Forty-one studies (21.5%) were published up to 2014, 36 (18.8%) between 2015 and 2018, 60 (31.4%) between 2019 and 2022, and 54 (28.3%) between 2023 and 2026. Overall, 114 studies (59.7%) were published from 2019 onwards. The temporal distribution of publications according to species is shown in Figure 2. Studies contributing equine data only among the target species predominated, accounting for 161 of the 191 included studies (84.3%). Twenty-nine studies (15.2%) contributed bovine data only, whereas one study (0.5%) included both bovine and equine data. Regarding study design, 81 studies (42.4%) were classified as in vivo, 59 (30.9%) as in vitro or ex vivo, and 51 (26.7%) as methodological studies. The PRP family was evaluated in 134 studies (70.2%), platelet lysate in 46 studies (24.1%), and platelet concentrate in 19 studies (9.9%). Because some studies evaluated more than one product family, these categories were not mutually exclusive. The distribution of clinical or application area and species is shown in Figure 3. Musculoskeletal applications represented the largest clearly classified clinical area, followed by reproductive applications. Bovine mammary disorders remained uncommon, with only five studies specifically addressing this field. Across the included studies, blood collection and platelet-based blood product preparation were performed using a wide range of approaches. Collection systems included evacuated tubes, syringes, transfusion bags, commercial closed systems, and plateletpheresis-based procedures. Preparation workflows comprised single- and double-centrifugation protocols, multi-step centrifugation, gravity-filtration systems, and apheresis-based approaches. Activation or lysis strategies included calcium-based activation, freeze–thaw cycles, chemical lysis, and protocols without exogenous activation, whereas storage and handling ranged from immediate use to refrigeration, freezing, and lyophilization. Overall, substantial methodological diversity was evident across the included studies and was not restricted to a single product family.

A quantitative summary of selected methodological characteristics contributing to this heterogeneity is provided in Table 2. Biological source varied substantially across studies, with autologous products used in 73 studies (38.2%) and allogeneic or donor-based products in 39 (20.4%), whereas source was not reported or remained unclear in 74 studies (38.7%). The jugular vein was the most frequently identifiable collection site (76/191; 39.8%), but the collection site was not reported or remained unclear in 108 studies (56.5%). Among explicitly identifiable anticoagulants, citrate/sodium citrate, ACD-based solutions, and CPD/CPDA-based solutions were all represented. Activation or lysis strategies included freeze–thaw or other physical lysis, calcium- or thrombin-based activation, and multiple comparative approaches, whereas storage ranged from fresh or short-term non-frozen use to frozen, cryopreserved, and lyophilized preparations.

### 3.3. Reconstructability of the Main Preparation Workflow

The availability of information needed to reconstruct the main blood collection and preparation workflow is summarized in Table 3. The main workflow was reconstructable in 111 studies (58.1%) and partially reconstructable in 80 studies (41.9%). These categories describe the information available on the main workflow rather than completeness across all methodological domains. All included studies met the minimum methodological information requirements specified in the eligibility criteria. A detailed study-level overview of blood collection, anticoagulant use, preparation workflow, activation or lysis, storage, product characterization, sterility or microbiological quality control, and administration is provided in Supplementary Table S2.

**Table 3:** Availability of information required to reconstruct the main blood collection and platelet-based blood product preparation workflow across the included studies.

Methodological Reporting Level	Definition	*n*	%
Reconstructable main workflow	Sufficient information was available to reconstruct the main blood collection and preparation workflow, including product identity, blood source or collection system, anticoagulant or an identifiable commercial or cited preparation protocol, principal processing steps, and final experimental or clinical use. Missing secondary numerical parameters did not preclude this classification when the overall workflow remained identifiable.	111	58.1
Partially reconstructable main workflow	One or more core elements required to reconstruct the main workflow could not be recovered with sufficient certainty from the article or from an explicitly identifiable cited or commercial protocol.	80	41.9
Total included in methodological synthesis		191	100.0

Note: Reconstructability refers to the main workflow and does not imply complete reporting of all procedural parameters or establish the methodological quality of the study. Reporting of individual methodological domains is presented separately in Table 4.

**Table 4:** Methodological reporting gaps across included studies. Frequency of reporting of key methodological items related to blood collection, platelet-based blood product preparation, product characterization, storage, and sterility or microbiological quality control across the 191 included studies.

Reporting Item	Applicable Studies (*n*)	Reported *n* (%)	Partially Reported *n* (%)	Not Reported *n* (%)	Not Applicable *n* (%)
Blood collection volume	191	67 (35.1)	4 (2.1)	120 (62.8)	0 (0.0)
Collection site	191	82 (42.9)	1 (0.5)	108 (56.5)	0 (0.0)
Anticoagulant	191	93 (48.7)	8 (4.2)	90 (47.1)	0 (0.0)
Centrifugation force/time	187	75 (40.1)	37 (19.8)	75 (40.1)	4 (2.1)
Platelet concentration	191	102 (53.4)	3 (1.6)	86 (45.0)	0 (0.0)
Leukocyte concentration	191	53 (27.7)	8 (4.2)	130 (68.1)	0 (0.0)
Activation/lysis method	191	50 (26.2)	28 (14.7)	113 (59.2)	0 (0.0)
Storage condition	191	65 (34.0)	2 (1.0)	124 (64.9)	0 (0.0)
Sterility or microbiological quality control	191	7 (3.7)	1 (0.5)	183 (95.8)	0 (0.0)

Note: Percentages for “reported”, “partially reported”, and “not reported” were calculated using the number of studies for which each methodological domain was considered applicable. Percentages for “not applicable” were calculated on the total number of included studies (*n* = 191). Studies classified as “not applicable” were excluded from the denominator used for the other three reporting categories. “Reported” indicates that the item was explicitly described and usable for methodological comparison. “Partially reported” indicates that the item was described incompletely, indirectly, or through reference to a commercial kit or previously published protocol but remained partially interpretable. “Not reported” indicates that the domain was applicable but the article did not provide interpretable information on the item. “Not applicable” was assigned only when the study design or preparation workflow clearly indicated that the methodological item did not apply. An explicit statement that a procedure was not performed was considered reported information rather than not applicable. For sterility or microbiological quality control, only explicit product-level microbiological or sterility assessment was considered reported; aseptic handling, diagnostic bacteriology unrelated to product quality control, and antimicrobial activity testing were not considered equivalent to sterility testing.

Blood collection methods included jugular vein collection, mammary vein collection, and other or unspecified collection sites. Collection systems included vacutainer tubes, syringes, blood transfusion bags, commercial closed systems, and automated plateletpheresis systems. Preparation methods included single-step centrifugation, double-centrifugation protocols, sedimentation-based methods, commercial kit-based processing, plateletpheresis, buffy-coat-based workflows, filtration, freeze–thaw procedures, and lyophilization.

### 3.4. Methodological Reporting Gaps

Methodological reporting gaps across the included studies are summarized in Table 4. For most domains, the methodological item was considered applicable to all 191 studies. Centrifugation parameters were considered not applicable in four studies in which the reported preparation workflow was based on non-centrifugal filtration or related non-centrifugal approaches, resulting in 187 applicable studies for this domain. Activation or lysis was considered applicable to all 191 studies.

Blood collection volume was explicitly reported in 67 studies (35.1%), collection site in 82 studies (42.9%), and anticoagulant use in 93 studies (48.7%). Among the 187 studies for which centrifugation parameters were applicable, centrifugation force and/or time were reported in 75 studies (40.1%), partially reported in 37 studies (19.8%), and not reported in 75 studies (40.1%).

Platelet concentration was reported in 102 studies (53.4%), whereas leukocyte concentration was reported in 53 studies (27.7%). Activation or lysis was reported in 50 studies (26.2%), partially reported in 28 studies (14.7%), and not reported in 113 studies (59.2%).

Storage conditions were reported in 65 studies (34.0%), while product-level sterility or microbiological quality control was reported in only 7 studies (3.7%) and partially reported in 1 study (0.5%).

Overall, the most pronounced reporting gaps concerned sterility or microbiological quality control, leukocyte characterization, activation or lysis procedures, blood collection volume, and storage conditions. Separating non-applicable items from unreported information avoided interpreting the absence of a procedural step as a reporting deficiency.

Stratified analyses showed that reporting completeness varied across species, study type, product family, and publication period. Among bovine studies, most collection and processing domains were more frequently reported than among equine studies, whereas leukocyte concentration was reported or partially reported in only 10.3% of bovine studies compared with 36.0% of equine studies. Methodological studies showed comparatively higher reporting of platelet and leukocyte concentrations, whereas no consistent improvement in reporting completeness was observed across publication periods. Product-specific patterns were also evident, particularly for activation or lysis, which was reported or partially reported in 95.7% of PL studies.

Reporting completeness was substantially higher among studies classified as having reconstructable workflows. In this group, anticoagulant information was reported or partially reported in 89.2% of studies and centrifugation parameters in 94.4% of applicable studies. Nevertheless, sterility or microbiological quality control remained infrequently reported even among reconstructable studies. Detailed stratified results are provided in Supplementary Table S3.

Product-level sterility or microbiological quality control also remained rarely reported in subgroups in which such information may be particularly relevant. It was reported or partially reported in 4 of 68 studies involving in vivo administration (5.9%), 3 of 43 studies using allogeneic or donor-based products (7.0%), 1 of 17 studies involving pooled products (5.9%), and 3 of 35 studies involving stored, frozen, or freeze-dried products (8.6%).

### 3.5. Focused Analysis of Studies on Bovine Mammary Disorders

The methodological and clinical characteristics of studies addressing bovine mammary disorders are summarized in Table 5. Five studies were included in this subset, representing 2.6% of the 191 studies included in the review.

Two studies investigated platelet concentrate, one investigated autologous PRP, and two investigated allogeneic P-PRP. Blood collection methods included autologous jugular collection, allogeneic jugular collection from healthy donor cows or heifers, and allogeneic collection from donor cows of the same farm via the subcutaneous mammary vein. CPDA-1 was the most frequently reported anticoagulant. The studies differed substantially in experimental unit and sample size, ranging from quarter-based designs to cow-level randomized clinical studies.

Preparation workflows included double centrifugation followed by freeze–thaw lysis for platelet concentrate, manual double centrifugation for autologous PRP, and centrifugation-based preparation followed by calcium gluconate activation for P-PRP. Reported storage conditions included frozen storage, on-ice maintenance until use, and fresh preparation before administration. All products in this subset were administered by the intramammary route. Administration protocols also varied, including single administration at dry-off and repeated administrations delivered at 12 or 24 h intervals or over multi-day treatment schedules.

The reported comparators included intramammary antibiotics, combined antibiotic and platelet-based blood product treatment, untreated controls, and alternative treatment groups. Outcome domains included clinical response, bacteriological cure, somatic cell count, milk amyloid A, inflammatory markers, relapse, and pathogen-specific response. Follow-up periods and outcome definitions were not uniform across studies, particularly for bacteriological cure and recurrence. Product-level sterility or microbiological quality control was explicitly reported in only one of the five studies.

Overall, the five studies investigated technically distinct platelet-based blood products and differed in clinical condition, experimental design, administration schedule, comparator treatment, follow-up, and outcome assessment. Therefore, they should not be interpreted as repeated evaluations of a single standardized intramammary platelet-based intervention, and their clinical findings were not quantitatively pooled or directly compared as estimates of treatment efficacy.

## 4. Discussion

The present systematic scoping review describes the literature on platelet-based blood products in bovine and equine medicine, with particular attention to blood collection and product preparation methods and with a focused analysis of bovine mammary disorders. Overall, 191 studies were included in the descriptive and methodological synthesis. Four main findings emerged from the review: the predominance of equine studies, the marked heterogeneity of collection and preparation workflows, the incomplete reporting of several key methodological domains, and the limited number of studies addressing bovine mammary applications.

The predominance of equine studies was marked, with studies contributing equine data only among the target species accounting for 161 of the 191 included studies (84.3%), whereas 29 studies (15.2%) contributed bovine data only and one study (0.5%) included both bovine and equine subjects. This pattern is consistent with the broader development of regenerative and orthobiologic approaches in equine medicine, particularly in musculoskeletal practice, where platelet-rich plasma and related products have been explored for tendon, ligament, and joint disorders for more than a decade [3,4]. By contrast, bovine applications remain comparatively limited, despite the clinical and production relevance of this species. This imbalance is particularly relevant in the context of the present review because the focused analysis of bovine mammary applications was included as a specific review objective. Mammary applications accounted for only a small proportion of the total corpus, although mastitis and other mammary disorders are among the most important health problems in dairy cattle and have major implications for animal health, productivity, and antimicrobial use [10,11,12,13].

The central finding of the review concerns the marked variability in blood collection and platelet-based blood product preparation. Differences were observed at almost every step of the workflow, including donor or patient selection, autologous versus allogeneic source, anatomical site of blood collection, blood volume, collection system, anticoagulant, centrifugation workflow, activation or lysis strategy, storage conditions, and administration protocol. This variability is relevant because platelet-based blood products are not biologically neutral or interchangeable formulations. Their cellular composition, mediator content, leukocyte contamination, and stability may all be influenced by how they are collected, processed, and handled. Accordingly, products grouped under the same broad term, especially PRP, may differ substantially from one study to another [1,2]. The observed variability likely reflected both genuine differences in preparation workflows and incomplete reporting of key procedural details. The present review did not formally quantify the relative contribution of these two sources of heterogeneity. These two aspects should be distinguished, because a protocol may be technically different but well described, whereas incomplete reporting may obscure whether apparently different procedures are truly distinct.

Heterogeneity was evident both before and after product preparation. Blood collection was performed using different anatomical sites and systems, ranging from tubes and syringes to transfusion bags, commercial closed systems, and automated plateletpheresis. These choices may influence platelet recovery, leukocyte and red blood cell contamination, activation status, and sterility risk, but they also reflect the practical realities of large-animal veterinary medicine, where procedures often need to be adapted to farm, field, or ambulatory conditions. Similarly, processing workflows differed in the number of centrifugation steps, relative centrifugal force, centrifugation time, temperature, fraction retained, buffy coat handling, activation or lysis method, filtration, lyophilization, and storage strategy. Therefore, the available literature describes a group of related platelet-based approaches rather than a single standardized intervention or preparation family.

A further relevant finding is the incompleteness of methodological reporting. By design, all 191 included studies met the minimum methodological-information requirements specified in the eligibility criteria. The main blood collection and preparation workflow was reconstructable in 111 studies (58.1%), whereas 80 studies (41.9%) had a partially reconstructable main workflow. These categories describe the availability of information needed to reconstruct the main workflow and should not be interpreted as a measure of overall methodological quality or complete reporting across all domains. The stratified analysis supported the internal consistency of this distinction: anticoagulant information and centrifugation parameters were reported or partially reported substantially more often in studies classified as reconstructable than in those classified as partially reconstructable. The reporting-gap analysis showed that key items were inconsistently described, particularly leukocyte concentration, activation or lysis procedures, storage conditions, and sterility or microbiological quality control. Incomplete reporting limits reproducibility and makes it difficult to determine whether differences in outcomes are attributable to clinical context, patient characteristics, or differences in the platelet-based blood product itself. This problem has long been recognized in the platelet concentrate literature, where classification systems have been proposed to reduce ambiguity and improve comparability among products with different cellular composition and preparation workflows [6,7].

The limited reporting of leukocyte concentration also deserves specific attention. Leukocyte concentration was reported or partially reported in only a minority of studies overall, and in only 10.3% of bovine studies compared with 36.0% of equine studies. Because leukocyte content can influence the inflammatory and biological profile of platelet-based preparations, incomplete characterization limits product comparability and mechanistic interpretation. This limitation is particularly relevant when comparing studies in musculoskeletal applications, including intra-articular and intratendinous use, where leukocyte-rich and leukocyte-poor preparations may not represent biologically equivalent interventions. However, the present review does not allow conclusions regarding the direction or magnitude of any effect of leukocyte content on clinical outcomes.

The limited reporting of sterility or microbiological quality control deserves specific attention. Product-level sterility or microbiological quality control was explicitly reported in only 7 studies (3.7%) and partially reported in one study (0.5%). Therefore, the present review can conclude that microbiological testing was rarely reported, but not necessarily that it was rarely performed. This distinction is important because absence of reporting does not always indicate absence of testing. Nevertheless, the lack of explicit information represents a relevant limitation for interpretation and reproducibility. This gap is particularly relevant for products intended for intramammary, intra-articular, intrauterine, or other applications where contamination could have important clinical consequences. It is also relevant for allogeneic, pooled, stored, frozen, or freeze-dried products, where preparation and handling involve additional steps and potentially longer intervals between collection and administration. In the present dataset, sterility or microbiological quality control was reported or partially reported in only 5.9% of studies involving in vivo administration, 7.0% of studies using allogeneic or donor-based products, 5.9% of studies involving pooled products, and 8.6% of studies involving stored, frozen, or freeze-dried products. These figures describe reporting frequency only and should not be interpreted as direct estimates of microbiological risk or product safety.

The focused analysis of bovine mammary studies illustrates the consequences of this heterogeneity in a clinically relevant subset. Only five studies addressing bovine mammary disorders were identified, and these did not investigate a uniform product. The subset included platelet concentrate, autologous PRP, and allogeneic pure PRP, obtained through different donor selection, blood collection, processing, activation, and storage workflows. Thus, although all five studies can be grouped under the broad category of platelet-based blood products for mammary disorders, they did not evaluate the same intervention in technical terms. The available evidence should therefore not be interpreted as repeated evaluations of one standardized platelet-based treatment, but rather as evaluations of distinct platelet-based preparations applied to a similar clinical target. Comparability was further limited by differences in clinical condition, pathogen profile, treatment timing, comparator therapy, outcome definition, and follow-up duration. Therefore, heterogeneity in this subset was not limited to product preparation but also involved study design and clinical context. The value of examining this subset lies primarily in identifying how platelet-based products have been prepared and reported for intramammary use and in highlighting the methodological information required for future comparable studies, rather than in estimating a pooled treatment effect.

Based on the reporting gaps observed in this review, we propose a set of review-informed reporting items for consideration in future studies. These items are intended to support more transparent reporting of platelet-based blood product preparation and use in bovine and equine medicine, rather than to define a validated consensus checklist. Depending on study design and intended product use, studies should clearly report donor or patient characteristics, blood collection site and volume, collection system, anticoagulant type and ratio, time from collection to processing, centrifugation parameters, fraction retained, final product volume, platelet and leukocyte concentrations, activation or lysis method, storage conditions, sterility testing, administration protocol, comparator treatments, and outcome assessment. For intramammary applications specifically, pathogen identification, bacteriological cure definition, somatic cell count assessment, treatment timing, dose and volume per quarter, number of administrations, and follow-up duration should also be reported. The proposed reporting items summarized in Table 6 are review-informed recommendations derived from the reporting gaps identified in the included literature and may help improve reproducibility, transparency, and comparability across studies while allowing technically justified differences among preparation protocols. These recommendations are intended to complement, rather than replace, existing platelet-product classification systems, which primarily facilitate classification according to product composition and preparation characteristics [6,7]. The present recommendations extend this perspective by emphasizing reporting of blood collection, processing, storage, microbiological quality control, administration, and outcome assessment in the specific context of bovine and equine studies.

The present review should be interpreted within the scope of its design. It was intentionally focused on bovine and equine medicine to provide a species-specific overview of platelet-based blood products in large-animal practice and to allow a focused analysis of bovine mammary studies. The search included Scopus, Web of Science Core Collection, CAB Abstracts, and PubMed but did not include grey literature or non-English publications, which may have resulted in missed studies. In addition, fibrin- or gel-based preparations, including PRF, were outside the analytical scope when the corresponding liquid platelet-based product could not be separately characterized or evaluated; therefore, the conclusions of this review should not be extended to these preparations. Although the search strategy included the main platelet-product terms and broader related expressions, not all specific product labels subsequently grouped within the PRP family were included as separate search terms, which may have reduced sensitivity for studies indexed only under those labels. This issue may be particularly relevant because most of the excluded non-English reports involved equine studies and PRP or PRP-type preparations, including both methodological studies and experimental applications. Accordingly, the English-language restriction may have resulted in some underrepresentation of the equine PRP literature. However, the direction of its effect on the reporting-completeness estimates cannot be determined without formal extraction and analysis of the excluded studies, because their methodological reporting could have been either more or less complete than that of the included English-language literature. In addition, the final database searches were conducted on 25 August 2026; therefore, the 2026 publication count represents a partial calendar year. No formal critical appraisal or risk-of-bias assessment was conducted, in line with the descriptive purpose of the scoping review. No formal review protocol was registered or published. The structured charting form and methodological variables were established and pilot-tested before full data extraction; however, the binary classification of the main workflow as reconstructable or partially reconstructable was formalized during the synthesis stage after study-level extraction and was subsequently applied systematically using explicit decision rules. In addition, eligibility required sufficient information to classify the platelet-based blood product and extract at least one methodological variable. This eligibility requirement may have preferentially retained studies providing at least minimal methodological information and may therefore have led to conservative estimates of reporting gaps relative to the broader literature. Therefore, the reporting-gap estimates apply to the included evidence base and should not be interpreted as representing all publications that merely mention the use of a platelet-based blood product. Because the review included heterogeneous products, study designs, clinical indications, and outcome definitions, quantitative pooling of clinical effects was not considered appropriate. Finally, although domain-specific applicability was considered explicitly in the reporting-gap analysis, some methodological domains may still differ in relevance across study designs and product types, and the small number of studies identified in the bovine mammary subset restricts the breadth of conclusions that can be drawn for this specific application area.

## 5. Conclusions

This systematic scoping review identified 191 studies investigating platelet-based blood products in bovine and equine medicine and showed that the field is broad but methodologically heterogeneous. Equine studies clearly predominated, while bovine applications remained comparatively limited. PRP and related PRP-type preparations represented the most frequently investigated product family, although platelet lysate and platelet concentrates were also represented. Considerable variability was observed in blood collection, anticoagulation, centrifugation, product characterization, activation or lysis, storage, and administration procedures. Methodological reporting was also frequently incomplete. The main preparation workflow was reconstructable in 111 studies (58.1%), and several key variables, particularly leukocyte concentration, activation or lysis procedures, storage conditions, and sterility or microbiological quality control, were inconsistently reported. Bovine mammary applications remained particularly limited, with only five studies identified. These studies evaluated technically distinct platelet-based blood products in different clinical contexts and therefore cannot be considered repeated evaluations of a single standardized treatment. Current evidence is consequently insufficient to define a standardized platelet-based intervention for bovine mammary disorders or to determine its comparative clinical effectiveness or antimicrobial-sparing potential. Future research should prioritize transparent reporting, consistent product characterization, and explicit microbiological quality control rather than assuming interchangeability between platelet-based preparations. Improved methodological reporting will be essential to strengthen reproducibility, facilitate comparisons among studies, and support the clinical translation of platelet-based blood products in large-animal veterinary medicine.

## Figures and Tables

**Table 1:** Descriptive summary of publication period, species, study type, and product family across the included studies.

Variable	Category	*n*	%
Publication period	≤2014	41	21.5
	2015–2018	36	18.8
	2019–2022	60	31.4
	2023–2026	54	28.3
Species	Equine	161	84.3
	Bovine	29	15.2
	Bovine and equine	1	0.5
Study type	In vivo	81	42.4
	In vitro or ex vivo	59	30.9
	Methodological	51	26.7
Product family *	PRP family	134	70.2
	PL	46	24.1
	PC	19	9.9

Abbreviations: ACP, autologous conditioned plasma; PC, platelet concentrate; PL, platelet lysate; P-PRP, pure platelet-rich plasma; PRP, platelet-rich plasma. * Product-family categories were not mutually exclusive because individual studies could investigate more than one platelet-based blood product. For aggregate analyses, P-PRP, ACP, PRGF, freeze-dried PRP, and other related PRP-type preparations were included within the broader PRP family when appropriate. Product-family assignment was based on the distinct preparation actually evaluated, characterized, or administered; upstream intermediates used only to generate a final product were not counted as additional product families. Freeze–thaw treatment alone did not result in reclassification as PL unless the resulting lysate or platelet extract was explicitly identified and evaluated as a distinct preparation. Product family was considered separately from biological source, activation or lysis, and storage or handling characteristics.

**Table 2:** Quantitative summary of selected methodological characteristics across the 191 included studies.

Methodological Characteristic	Category	*n*	%
Biological source	Autologous	73	38.2
	Allogeneic/donor-based	39	20.4
	Mixed/comparative	5	2.6
	Not reported/unclear	74	38.7
Blood collection site	Jugular vein	76	39.8
	Subcutaneous mammary vein	4	2.1
	Other specified site	2	1.0
	Partially specified	1	0.5
	Not reported/unclear	108	56.5
Anticoagulant family	Citrate/sodium citrate	32	16.8
	ACD-based	27	14.1
	CPD/CPDA-based	27	14.1
	Multiple/comparative/other specified	7	3.7
	Not reported/unclear	98	51.3
Activation/lysis	Freeze–thaw/physical lysis	29	15.2
	Calcium- and/or thrombin-based activation	10	5.2
	Multiple/comparative methods	8	4.2
	Other specified approach	3	1.6
	No exogenous activation/lysis	5	2.6
	Lysis reported; method not specified	23	12.0
	Not reported	113	59.2
Storage/handling	Fresh/immediate or short-term non-frozen	19	9.9
	Frozen/cryopreserved	29	15.2
	Freeze-dried/lyophilized	3	1.6
	Multiple/comparative storage conditions	13	6.8
	Other/partially specified	3	1.6
	Not reported	124	64.9

Note: Percentages were calculated using all included studies (*n* = 191) as the denominator. Categories within each methodological characteristic were mutually exclusive. Anticoagulant categories were grouped according to the anticoagulant family explicitly reported in the study-level extraction; studies reporting multiple or comparative anticoagulants were grouped separately. “Other specified site” included explicitly reported anatomical collection sites other than the jugular or subcutaneous mammary vein. Activation/lysis and storage categories were grouped descriptively from the study-level information reported in Supplementary Table S2.

**Table 5:** Methodological and clinical characteristics of studies addressing bovine mammary disorders.

Study	Condition/Sample	Product/Source	Main Preparation Features	Dose/Administration	Comparator(s)/Allocation	Follow-Up and Outcomes	Product Sterility/QC
Lange-Consiglio et al., 2014 [14]	Acute and chronic clinical mastitis; 229 mastitic quarters from three farms	PC; allogeneic within farm	Double centrifugation; platelet concentration standardized; freeze–thaw treatment	5 mL intramammary per affected quarter; treatment for 3 consecutive days	Antibiotic; PC; antibiotic + PC; allocation method and blinding NR	Clinical response, SCC, and relapse; exact follow-up interval for relapse NR	NR; milk bacteriology was used for mastitis diagnosis and was not considered product-level sterility testing
Evkuran Dal et al., 2019 [15]	Subclinical mastitis; 120 mammary quarters (40 per treatment group)	PC; allogeneic	Double centrifugation; platelet concentration standardized; freeze–thaw treatment	5 mL intramammary via teat catheter; repeated at 3-day intervals over a 12-day treatment period	Antibiotic; PC; antibiotic + PC; random allocation reported; blinding NR	Milk evaluated on days 0, 7, 14, and 21; SCC, milk amyloid A, treatment response, and recurrence	NR; aseptic administration was not considered equivalent to product-level microbiological QC
Terra et al., 2020 [16]	Subclinical mastitis at dry-off; 36 quarters initially enrolled, 34 analyzed from 9 cows	PRP; autologous	Manual double centrifugation	10 mL intramammary per quarter at dry-off	Dry-cow antibiotic; PRP; PRP + antibiotic; completely randomized design; blinding NR	Assessment before dry-off, at calving, and at 14, 30, and 60 days postpartum; SCC and milk amyloid A	Reported; microbiological culture of a PRP aliquot showed no growth
Duque-Madrid et al., 2021 [17]	Gram-positive subclinical mastitis; 103 cows (54 P-PRP, 49 cefquinome)	P-PRP; allogeneic	Centrifugation-based preparation; calcium gluconate activation	10 mL intramammary; three administrations at 12 h intervals; persistent cases retreated at day 26	Cefquinome; random allocation; blinding NR	Primary bacteriological cure assessed on days 21–22; second assessment on days 47–48 after retreatment when required. Cure defined as absence of the baseline organism in two post-treatment samples; SCC and inflammatory mediators also evaluated	NR; sterile preparation/handling was not considered product-level sterility testing
López et al., 2024 [18]	Gram-positive subclinical mastitis; 130 cows completing the study (40 P-PRP, 28 cloxacillin, 36 P-PRP + cloxacillin, 26 untreated)	P-PRP; allogeneic	Centrifugation-based preparation; calcium gluconate activation	10 mL intramammary; three administrations at 24 h intervals on three consecutive days	Cloxacillin; P-PRP; P-PRP + cloxacillin; untreated control; randomized clinical trial; treatment administration blinded	22-day follow-up; post-treatment sampling on days 21 and 22. Bacteriological cure defined as absence of the baseline organism from both post-treatment cultures; SCC and inflammatory/growth mediators also assessed	NR

Abbreviations: PC, platelet concentrate; PRP, platelet-rich plasma; P-PRP, pure platelet-rich plasma; SCC, somatic cell count; NR, not reported. Note: Product sterility/QC refers specifically to microbiological quality control performed on the platelet-based product; diagnostic milk bacteriology and aseptic handling alone were not considered equivalent to product-level sterility testing. For Evkuran Dal et al. [15], the full Methods describe treatment at 3-day intervals over 12 days, whereas the abstract refers more generally to treatment “for 3 days”; the schedule reported in the table follows the Section 2 (Materials and Methods).

**Table 6:** Review-informed reporting recommendations for future studies using platelet-based blood products in bovine and equine medicine. Recommendations are based on the methodological gaps identified across the included studies and are not intended as a validated consensus checklist.

Methodological Domain	Priority	Recommended Reporting Items	Rationale
Donor or patient selection	Essential	Species, breed, age, sex, health status, reproductive or lactation status when relevant, inclusion/exclusion criteria, recent drug treatments, and number of donors or treated animals.	Donor characteristics may influence platelet concentration, leukocyte content, growth factor profile, and product consistency.
Blood collection	Essential	Collection site, needle or catheter size, blood volume collected, collection system, bag or tube type, anticoagulant type and volume, time from collection to processing, and transport temperature.	These variables may affect platelet recovery, activation status, leukocyte contamination, sterility risk, and reproducibility of the protocol.
Anticoagulant and collection system	Essential	Exact anticoagulant used, anticoagulant-to-blood ratio, commercial kit or bag system, manufacturer, and whether the system is open or closed.	Anticoagulant type and collection system may influence platelet quality and contamination risk, especially for allogeneic or stored products.
Processing protocol	Essential	Number of centrifugation steps, relative centrifugal force or rpm with rotor details, time, temperature, brake use, plasma fraction collected, buffy coat inclusion or exclusion, filtration, pooling, and final product volume.	Processing parameters may influence whether the final product is leukocyte-poor or leukocyte-rich, platelet concentration, and comparability between studies.
Product classification	Essential	Original product terminology and broader product family (e.g., PRP family, PC, or PL); relevant compositional characteristics such as leukocyte-poor or leukocyte-rich when available; and biological source (e.g., autologous, allogeneic, donor-based, or mixed/comparative). Commercial kit or proprietary preparation system, activation or lysis, lyophilization, and storage conditions should be reported separately in the corresponding methodological domains.	Standardized product classification is important for comparing biological and clinical outcomes across studies.
Product characterization	Essential	Platelet concentration, fold increase over whole blood, leukocyte concentration, red blood cell contamination, mean platelet volume when available, and relevant growth factors or cytokines.	Biological activity is difficult to interpret without minimal characterization of platelet and leukocyte content.
Activation or lysis	Essential	Whether the product was activated or lysed; activating agent or lysis method; dose or ratio; timing relative to administration; number of freeze–thaw cycles when used.	Activation and lysis may modify the release of platelet-derived mediators and could affect biological or clinical responses.
Storage and handling	Essential	Fresh use or storage; storage temperature, duration, freeze–thaw conditions, thawing method, lyophilization procedure when applicable, and time between preparation and administration.	Storage conditions may influence platelet integrity, mediator availability, sterility, and product stability.
Sterility or microbiological quality control	Essential for products intended for administration; desirable for purely analytical/in vitro studies	Sterility testing, aerobic/anaerobic culture, mycoplasma or endotoxin testing when relevant, timing of testing, number of tested aliquots, and criteria for product exclusion. For products intended for administration, report whether microbiological testing was performed before release or use and the acceptance or rejection criteria.	Safety reporting is particularly important for intramammary, intra-articular, intrauterine, allogeneic, pooled, or stored products.
Administration protocol	Essential when administered	Route, anatomical site, dose volume, number of administrations, interval between treatments, use of image guidance when relevant, and whether the product was combined with antibiotics, cells, or other therapies.	Clinical interpretation requires a clear description of how and when the product was delivered.
Comparator and co-interventions	Essential for comparative studies	Control group, placebo or standard therapy, antimicrobial use, concomitant treatments, and withdrawal or exclusion of additional therapies.	Comparator choice and co-interventions may influence the interpretation of efficacy and antimicrobial-sparing potential.
Outcomes and follow-up	Essential for biological/clinical studies	Primary and secondary outcomes, timing of assessment, clinical, microbiological, inflammatory, imaging, or production-related endpoints, and follow-up duration. For intramammary studies, also report pathogen identification, the definition of bacteriological cure, SCC assessment, dose and volume per quarter, and the number and timing of administrations.	Outcome heterogeneity limits synthesis; clear outcome reporting improves comparability and interpretation.
Reproducibility and quality control	Desirable and essential when manufacturing performance or scalability is an objective	Batch-to-batch variability, number of preparations, failed preparations, platelet recovery, sterility failures, product rejection criteria, and operator or laboratory standardization.	Quality-control reporting is necessary to evaluate feasibility, scalability, and transferability to field conditions.

Abbreviations: PC, platelet concentrate; PL, platelet lysate; PRP, platelet-rich plasma. Note: These recommendations are derived from reporting gaps identified in the present scoping review. They are intended to support transparent and reproducible reporting and should not be interpreted as a validated checklist or formal consensus standard. Items identified as essential only under specific conditions apply when relevant to the study design, product use, or experimental objective. Some recommended items extend beyond the nine domains quantitatively assessed in the reporting-completeness analysis and reflect methodological information identified during study-level charting and author interpretation. The quantitatively assessed domains were blood collection volume, collection site, anticoagulant, centrifugation force/time, platelet concentration, leukocyte concentration, activation or lysis, storage, and sterility or microbiological quality control. Other recommended items in Table 6 were derived from study-level charting and author interpretation rather than from the quantitative reporting-gap analysis. These recommendations should therefore be regarded as review-informed rather than as empirically validated or consensus-based reporting standards.

## Data Availability

The original contributions presented in this study are included in the article/Supplementary Materials. Further inquiries can be directed to the corresponding authors.

## Supplementary Materials

The following supporting information can be downloaded at: https://www.mdpi.com/article/10.3390/vetsci13101112/s1, Table S1, Full database search strategies used in the systematic scoping review; Table S2, Study-level methodological extraction of the 191 included studies on platelet-based blood products in bovine and equine medicine; Table S3, Stratified methodological reporting analyses across the 191 included studies; Table S4, Preferred Reporting Items for Systematic reviews and Meta-Analyses extension for Scoping Reviews (PRISMA-ScR) Checklist.

## Abbreviations

The following abbreviations are used in this manuscript:

ACP	autologous conditioned plasma;
CPDA-1	citrate-phosphate-dextrose-adenine;
N/A	not applicable;
NR	not reported;
PC	platelet concentrate;
PL	platelet lysate;
P-PRP	pure platelet-rich plasma;
PRISMA-ScR	Preferred Reporting Items for Systematic Reviews and Meta-Analyses extension for Scoping Reviews;
PRP	platelet-rich plasma;
SCC	somatic cell count;
SCM	subclinical mastitis
